# Adverse Responses following Exposure to Subtoxic Concentrations of Zinc Oxide and Nickle Oxide Nanoparticles in the Raw 264.7 Cells

**DOI:** 10.3390/toxics11080674

**Published:** 2023-08-06

**Authors:** Nasser B. Alsaleh, Mohammed A. Assiri, Anas M. Aljarbou, Mohammed M. Almutairi, Homood M. As Sobeai, Ali A. Alshamrani, Sultan Almudimeegh

**Affiliations:** Department of Pharmacology and Toxicology, College of Pharmacy, King Saud University, P.O. Box 2457, Riyadh 11451, Saudi Arabia; moassiri@ksu.edu.sa (M.A.A.); 442106265@student.ksu.edu.sa (A.M.A.); malmotyre@ksu.edu.sa (M.M.A.); hassobeai@ksu.edu.sa (H.M.A.S.); aaalshamrani@ksu.edu.sa (A.A.A.); salmudimeegh@ksu.edu.sa (S.A.)

**Keywords:** nanotechnology, engineered nanomaterials, nanotoxicity, environmental health, biomedical applications

## Abstract

The incorporation of engineered nanomaterials (ENMs) in biomedical and consumer products has been growing, leading to increased human exposure. Previous research was largely focused on studying direct ENM toxicity in unrealistic high-exposure settings. This could result in overlooking potential adverse responses at low and subtoxic exposure levels. This study investigated adverse cellular outcomes to subtoxic concentrations of zinc oxide (ZnONPs) or nickel oxide (NiONPs) nanoparticles in the Raw 264.7 cells, a macrophage-like cell model. Exposure to both nanoparticles resulted in a concentration-dependent reduction of cell viability. A subtoxic concentration of 6.25 µg/mL (i.e., no observed adverse effect level) was used in subsequent experiments. Exposure to both nanoparticles at subtoxic levels induced reactive oxygen species generation. Cellular internalization data demonstrated significant uptake of NiONPs, while there was minimal uptake of ZnONPs, suggesting a membrane-driven interaction. Although subtoxic exposure to both nanoparticles was not associated with cell activation (based on the expression of MHC-II and CD86 surface markers), it resulted in the modulation of the lipopolysaccharide-induced inflammatory response (TNFα and IL6), and cells exposed to ZnONPs had reduced cell phagocytic capacity. Furthermore, subtoxic exposure to the nanoparticles distinctly altered the levels of several cellular metabolites involved in cell bioenergetics. These findings suggest that exposure to ENMs at subtoxic levels may not be devoid of adverse health outcomes. This emphasizes the importance of establishing sensitive endpoints of exposure and toxicity beyond conventional toxicological testing.

## 1. Introduction

The development of nanotechnology and the use of engineered nanomaterials (ENMs), precisely synthesized materials within a size range of 1–100 nm in at least one dimension, has been rapidly growing over the past few years [1,2]. ENM applications are found across most industries, including energy, electronics, healthcare, and biotechnology [2,3,4]. Despite previous efforts, human and environmental safety of ENMs remains to be understood, and currently, there is limited regulation for the use of ENMs in consumer and medical products [1,3,4,5,6,7,8], possibly attributed to the uncertainty of ENM risks to human health and the environment [1,4,6,7,8]. One major challenge facing the scientific community in assessing the safety of ENMs is the vast number of existing and emerging ENMs [1,7]. Accumulated research over the past few years and continued efforts have proven critical for regulatory bodies, as evidenced by the latest regulation in France and the European Commission on the use of titanium dioxide nanoparticles as a food additive [5,9]. Nevertheless, more research is warranted to better predict and evaluate potential toxicities associated with exposure to ENMs.

Previous efforts were focused on studying and understanding direct and overt ENM toxicity on different experimental models, with the majority of studies being carried out at high and unrealistic exposure levels [1,7,10,11]. Although these studies are important to establish the fundamentals of nanotoxicity, including the extent of ENM toxicity (i.e., hazard), subtle adverse responses at low and subtoxic exposure levels could be overlooked. Indeed, exposure to ENMs (e.g., in biomedical and consumer products) typically occurs at low levels and over a long period of time [1,2,4,8]. Therefore, assessing potential adverse responses following exposure to low and subtoxic levels of ENMs is critical to ensure their long-term safety. It is worth noting that precise assessment of worker, consumer, and public exposure to ENMs remains a challenge due to several reasons, but most importantly, the assessment of nanomaterial release and the analytical limitations with regard to their physicochemical properties [12,13].

The immune system is a key system to studying ENM biointeractions, as one of its primary functions is to eliminate foreign substances, including ENMs [14]. Previous studies have demonstrated that ENM-induced immunotoxicity is primarily linked to its interaction with innate immune cells [14,15,16,17]. Such toxicity extends to multiple body systems, as innate immune cells are not confined to one organ but are found across most body tissues and they play key roles in regulating tissue homeostasis. Most importantly, we and others have previously shown that exposure to widely used inorganic ENMs, such as silver, iron oxide, and titanium dioxide, is associated with adverse immune responses, even at concentrations that did not result in apparent toxicity [15,17,18,19,20,21]. For instance, some of these adverse responses included disruption of key signaling pathways and cellular functions. Furthermore, previous findings have demonstrated that ENM-induced modulation of cellular function is not always suppressive in nature but can be stimulatory, or have both suppressive and stimulatory responses simultaneously [19,22,23]. Nevertheless, more research is warranted to better characterize the ENM biointeractions at subtoxic exposure levels, particularly ENMs that are known to induce toxicity at high exposure levels and are deemed safe at subtoxic concentrations, as per conventional toxicological assays (e.g., cell viability).

Macrophages are an important cell type involved in host protection against invading pathogens and foreign substances. With their wide tissue distribution, it is unsurprising that alteration to their function could lead to a wide range of pathologies, including cancer, cardiovascular and neurodegenerative diseases [24]. Macrophages also make up the reticuloendothelial system (RES), where they phagocytose foreign substances, including ENMs, mainly in the liver and spleen [25]. Therefore, macrophages represent a pivotal model for studying ENM biointeraction and toxicity [14,26]. Omics techniques (e.g., proteomics, metabolomics, lipidomics, etc.) have been revolutionizing several disciplines, including nanotoxicology [27,28]. Indeed, they provide an overview of the global changes in cell state following exposure to toxic insults [28]. Assessment of cell metabolomics (i.e., changes in the global level of metabolites) following exposure to ENMs could be a great tool to assess the safety of ENMs, unravel novel biomarkers, and understand the underlying molecular mechanisms of ENM toxicity [27,28]. In this study, we sought to investigate adverse cellular responses associated with exposure to subtoxic concentrations of nanoparticles in Raw 264.7 cells, a macrophage-like cell model. Specifically, we assessed several endpoints, including reactive oxygen species (ROS) generation, nanoparticle internalization, cell activation, functional capacity, and metabolomic changes following exposure to subtoxic concentrations of ZnONPs or NiONPs. The novelty of this study is to first identify subtoxic concentrations (i.e., concentrations that do not result in significant reduction of cell viability), and then, use these concentrations to assess potential adverse outcomes beyond direct toxicity endpoints. The rational of this work is that average human exposure to ENMs will be far below the concentrations that are used in the majority of nanotoxicological studies.

## 2. Materials and Methods

### 2.1. Nanoparticle Preparation and Characterization

Both nanoparticles were obtained from our collaborators, and stocks were prepared from fresh powder before carrying out biological experiments [29,30]. Briefly, the nanoparticles original stock with a concentration of 1 mg/mL was dispersed using a water-bath sonicator. Serial working stocks (dilutions) were made from the original stock to reach the desired final concentrations with consistent use of brief episodes of vortexing before treating cells. Hydrodynamic size (nm), zeta potential (mV), and polydispersity index (PDI) were measured for both types of nanoparticles in purified water and cell culture media using Zetasizer (Malvern, Westborough, MA, USA). Nanoparticle size and shape were qualitatively evaluated by a transmission electron microscope (TEM). Briefly, samples were sonicated for 10 min, and then one drop was poured upon the grid (Copper, Ted Pella, 300 mesh) and let dry overnight. Samples were visualized, and images were captured using JEOL JEM1010 TEM (Japan) at an operating voltage of 80 kV.

### 2.2. Cell Culture

Raw 264.7 cells (TIB-71TM) were purchased from the American Type Culture Collection (ATCC, Manassas, VA, USA) and cultured according to ATCC guidelines. Briefly, cells were cultured at 37 °C and 5% CO_2_ following standard cell culture aseptic procedures. Cells were cultured in Dulbecco’s Modified Eagle’s Medium (DMEM) supplemented with 10% fetal bovine serum (FBS) and 100 U penicillin/mL: 100 µg streptomycin/mL. Cells were subcultured and used up to 15 passages throughout the experiments.

### 2.3. Measuring Cell Viability

Cell viability was measured based on the formation of colored formazan crystal from MTT (3-(4,5-dimethylthiazol-2-yl)-2,5-diphenyltetrazolium bromide) (Tokyo Chemical Industry, Tokyo, Japan). Briefly, cells were grown in supplemented DMEM media in a 96-well plate until reaching ~70% confluency. Supplemented media was replaced with serum-free media, and then cells were treated with nanoparticles (1.56–100 µg/mL) for 24 h. MTT was solubilized in sterile PBS and added to cells (a final concentration of 500 µg/mL) for ~1–3 h or until blue formazan crystals formed. The supernatant was discarded and the crystals were dissolved in isopropyl alcohol (IPA). After placing the microplate on a shaker for 10 min, absorbance was measured by a spectrophotometer at a wavelength of 570 nm (Synergy HT system, BioTek, Winooski, VT, USA).

### 2.4. Assessment of ROS Formation

The formation of ROS was measured using dichlorofluorescin diacetate (H2DCFDA) (ThermoFisher Scientific, Waltham, MA, USA). Briefly, cells were grown in supplemented media in a 96-well microplate until reaching 70% confluency. Supplemented media was replaced with serum-free media, and then cells were treated with nanoparticles (1.56–6.25 µg/mL) for 24 h. Cells were washed with PBS and 5 mM H2DCFDA (in PBS) was then added to cells for 30 min at 37 °C, protected from direct light. Fluorescence was measured using a spectrophotometer at excitation/emission of 495/527 nm (Synergy HT system, BioTek, Winooski, VT, USA).

### 2.5. Nanoparticle Cellular Internalization

Nanoparticle uptake into cells was measured using inductively coupled plasma mass spectrometry (ICP-MS). Briefly, cells were grown in a 24-well plate until reaching 70% confluency. Supplemented media was replaced with serum-free media, and cells were then treated with nanoparticles at 6.25 µg/mL for 24 h. Cells were gently washed with ice-cold PBS and collected in Eppendorf tubes to wash off nanoparticles that were not internalized by the cells. Cell pellets were then dissolved in 70% HNO_3,_ after which samples were diluted to 1% HNO_3_ and nanoparticle metal content was quantified using Perkin Elmer “Elan” 9000 ICP-MS system (Waltham, MA, USA). An internal standard containing lithium (Li), yttrium (Y), and indium (In) was used. All metals were detected at a level of 0.05 ppb resolution.

### 2.6. Measuring Gene Expression

Gene expression was measured based on mRNA amplification using real-time PCR (Applied Biosystems, ThermoFisher Scientific, Waltham, MA, USA). Briefly, cells were grown in a 24-well plate until reaching 70% confluency. Supplemented media was replaced with serum-free media, and cells were then treated with nanoparticles at 6.25 µg/mL for 6 h. Cells were lysed using TRIzol Reagent (ThermoFisher Scientific, Waltham, MA, USA) and samples were kept at −80 °C until further processing. mRNA was isolated according to the TRI manufacturer’s instructions. The quantity and quality of isolated mRNA was measured by Nano-Drop 2000 system (ThermoFisher Scientific, Waltham, MA, USA). mRNA was then reverse transcribed into cDNA using MedChemExpress 2x Super RT Mix (Monmouth Junction, NJ, USA) and Eppendorf thermocycler (Hamburg, Germany). Real-time PCR was performed using MedChemExpress SYBR Green qPCR Master Mix (Low ROX) (Monmouth Junction, NJ, USA), and primers were purchased from Integrated DNA Technologies (Coralville, IO, USA). Differential gene expression was calculated using the relative quantification method (2^−∆∆Ct^). GAPDH was used to normalize target gene C_t_ values. Primer sequences were based on algorithm-generated sequences from Primer Bank: http://pga.mgh.harvard.edu/primerbank (accessed on 1 March 2023). TNFα forward, 5′-CCT GTA GCC CAC GTC GTA G-3′; TNFα reverse, 5′-GGG AGT AGA CAA GGT ACA ACC C-3′; IL-6 forward, 5′-CTG CAA GAG ACT TCC ATC CAG-3′; IL-6 reverse, 5′-AGT GGT ATA GAC AGG TCT GTT GG-3′; GAPDH forward, 5′-AGGATCCTTGAAGACCACCA-3′; GAPDH reverse, 5′-GAGTTGCTGTTGAAGTCGCA-3.

### 2.7. Cell Activation Makers

The expression of cell surface markers MHC-II and CD86 were measured using fluorescently tagged antibodies (BD Bioscience, Franklin Lakes, NJ, USA). Briefly, cells were grown in a 24-well plate until reaching 70% confluency. Supplemented media was replaced with serum-free media and cells were then treated with nanoparticles at 6.25 µg/mL for 24 h. Cells were then washed with PBS and incubated with the antibodies (1:100) for 1 h at room temperature. Cells were then washed 2X with PBS, and fluorescence was measured using BD Accuri^TM^ C6 flow cytometer (BD Bioscience, Franklin Lakes, NJ, USA). A minimum of 10,000 events were utilized to calculate the mean fluorescence intensity (MFI) of the samples.

### 2.8. Assessment of Cell Phagocytosis

Cell phagocytosis was measured based on the cellular uptake of latex beads coated with fluorophore-labeled rabbit IgG following the manufacturer’s instructions (Cayman, Ann Arbor, MI, USA). Briefly, cells were grown in a 24-well plate until reaching 70% confluency. The supplemented media was replaced with serum-free media, and cells were then treated with nanoparticles at 6.25 µg/mL for 24 h. Cells were then washed with PBS and exposed to the beads (1:100) for 4 h at 37 °C and 5% CO_2,_ after which, they were washed gently with PBS and collected in Eppendorf tubes. Fluorescence was measured using a BD Accuri^TM^ C6 flow cytometer (BD Bioscience, Franklin Lakes, NJ, USA). A minimum of 10,000 events were utilized to calculate the MFI of the samples.

### 2.9. Metabolomics Study

Cells were grown in a 6-well plate until reaching 70% confluency. Supplemented media was replaced with serum-free media and then the cells were treated with nanoparticles at 6.25 µg/mL for 24 h. Thereafter, the cells were washed with ice-cold PBS and then scraped with ice-cold HPLC-grade methanol. Samples were vortexed for 20 min and then centrifuged at 20,000× *g* at 4 °C for 10 min. The supernatant was isolated and vacuum dried. Methoxymation was carried out by adding 100 μL of methoxyamine HCl in a pyridine solution (20 mg/mL). Samples were derivatized by BSTFA/TMCS (99/1 *v*/*v*), and 1 μL of the derivatized sample was injected into the system with the split mode (split ratio = 1:20). Gas chromatography–mass spectroscopy (GC-MS) (Perkin Elmer Clarus 600) was used for the analysis. The Elite chromatographic 5MS column (30 m × 0.25 mm × 0.25 µm film thickness) was used, with high-purity helium at a flow rate of 1 mL/min. The injector temperature was 280 °C using a splitless injector at 20:1. The initial temperature was 40 °C (held for 1 min), which was then increased to 150 °C and finally increased to 300 °C (1 min; increased at 10 °C per minute). The range of the mass scanning was 40 to 600 at 70 eV electron energy. The peaks of the spectra were identified by the National Institute of Standard and Technology Library (NIST 2005) and the Wiley Library. Filtered data were sorted and uploaded to MetaboAnalyst 5.0. Data was normalized to pooled non-treated (NT) control samples, log-transformed, and auto-scaled before performing statistical and enrichment analyses.

### 2.10. Statistical Analysis

Data is presented as mean ± standard error of the mean (SEM). One-way analysis of variance (ANOVA) followed by Bonferroni post hoc analysis was performed for multi-group treatment studies, whereas Student’s *t*-test was utilized for two-group comparison. Statistical significance indicates a *p*-value of less than 0.05. GraphPad Prism 9 software was used for statistical analysis and graph generation (GraphPad Inc., San Diego, CA, USA).

## 3. Results

### 3.1. Nanoparticle Characterization

**Figure 1:**
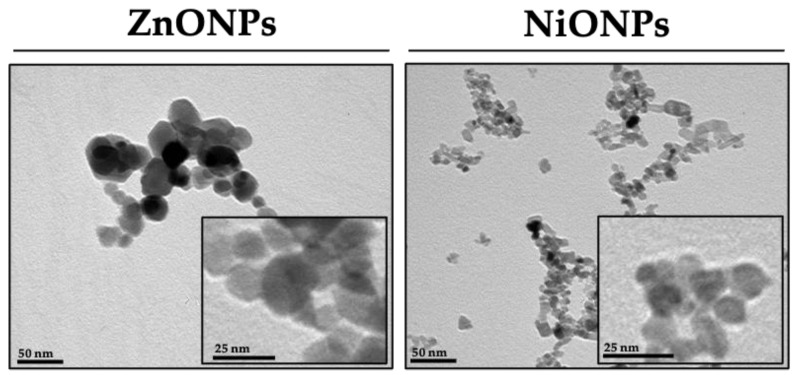
**Transmission electron microscopy images of the nanoparticles**. Representative transmission electron microscopy (TEM) images for ZnONPs (left panel) and NiONPs (right panel). The images qualitatively demonstrate the size and shape of each nanoparticle type at two different magnifications.

Nanoparticles were characterized by their size, shape, and surface charge. Representative transmission electron microscopy (TEM) images demonstrated the size and shape of the nanoparticles (approx. diameter size was 20 nm for ZnONPs and 15 nm for NiONPs) (Figure 1). Based on the dynamic light scattering (DLS) analysis, ZnONPs have a hydrodynamic size of 336.8 ± 25.5 nm in ddH_2_O (vehicle) and 427.7.8 ± 18.9 nm in cell culture media (DMEM), whereas NiONPs have a hydrodynamic size of 217.6 ± 11.3 nm in water and 384.7 ± 21.3 nm in cell culture media (Table 1). This indicates the tendency for the nanoparticles to aggregate in vehicle and DMEM, and hence, the nanoparticles were sonicated in a water-bath sonicator and vortexed well just before treatment. Furthermore, ZnONPs have a surface charge of −17.9 ± 2.1 mV in ddH_2_O and −1.5 ± 0.2 mV in cell culture media, whereas NiONPs have a surface charge of −36.3 ± 1.4 mV in ddH_2_O and −14.9 ± 1.9 mV in cell culture media (Table 1).

### 3.2. Cell Viability following Exposure to the Nanoparticles

**Figure 2:**
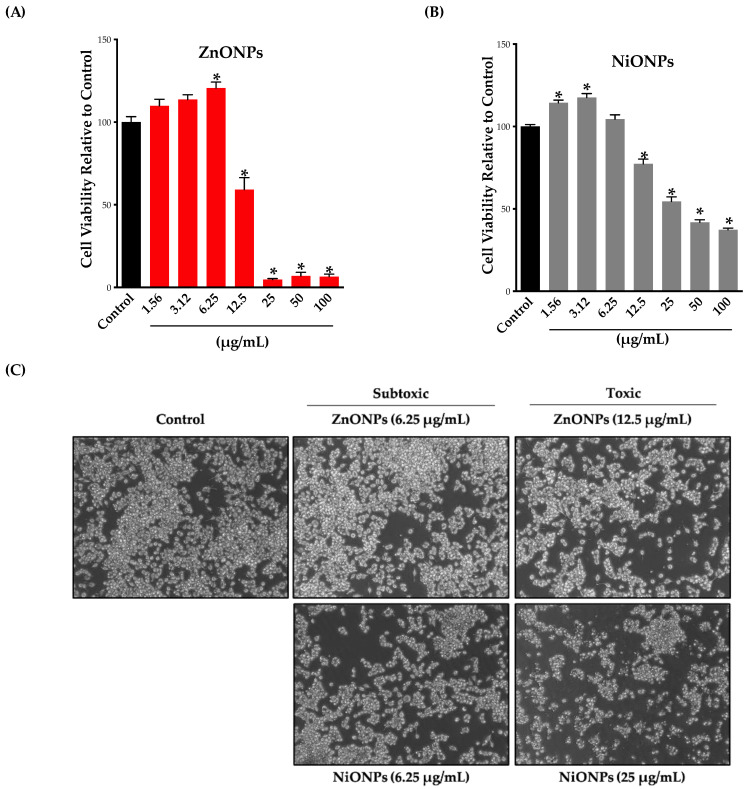
**Cell viability and morphology following exposure to nanoparticles.** (**A**,**B**) Cell viability was evaluated following exposure to nanoparticles using the MTT assay. Cells were exposed to (**A**) ZnONPs (red bars) or (**B**) NiONPs (grey bars) for 24 h in serum-free cell culture media at a concentration range of 1.56–100 µg/mL. (**C**) Cell morphological changes following exposure to nanoparticles were assessed by a phase-contrast microscope. Cells were exposed to ZnONPs or NiONPs for 24 h in serum-free cell culture media at a subtoxic concentration of 6.25 µg/mL and a toxic concentration of 12.5 µg/mL for ZnONPs and 25 µg/mL for NiONPs. Experiments were independently repeated at least three times (*n* ≥ 3), and statistical significance (*) indicates the difference between the treatment groups (black bars) with *p*-values equal to or smaller than 0.05 (*p* ≤ 0.05).

To assess cell viability following exposure to ZnONPs or NiONPs, we exposed Raw 264.7 cells to the nanoparticles at a concentration range of 1.56–100 µg/mL for 24 h and then evaluated cell viability based on the cell mitochondrial activity using the MTT assay. Our results showed that exposure of Raw 264.7 cells to ZnONPs or NiONPs resulted in reduced viability at concentrations of 12 µg/mL or higher, with the ZnONPs being more cytotoxic compared to NiONPs (Figure 2A,B). Furthermore, our data demonstrated a sharp drop in viability at 12 µg/mL in ZnONP-treated cells with almost complete loss of viability at higher concentrations (i.e., >90% reduction of viability) (Figure 2A). However, exposure to NiONPs resulted in a gradual decrease in cellular viability over the tested range of concentrations (Figure 2B). Based on the viability results, we chose a concentration of 6.25 µg/mL as our subtoxic concentration or no observed adverse effect level (NOAEL) for both nanoparticle types. This concentration was used in all subsequent experiments.

We sought to confirm the viability assay results qualitatively at the cell morphological level. To do so, we exposed the cells to toxic (ZnONPs, 12.5 µg/mL; NiONPs, 25 µg/mL) and subtoxic (6.25 µg/mL for both ZnONPs and NiONPs) concentrations of the nanoparticles for 24 h and then evaluated cellular morphology by phase-contrast microscopy (Figure 2C). Notice that we used a concentration of 12.5 µg/mL for ZnONPs and 25 µg/mL for NiONPs since both produced ~50% reduction of viability. Our results confirmed that cell exposure to ZnONPs or NiONPs at 6.25 µg/mL was not associated with morphological changes; however, at higher concentrations, both nanoparticles resulted in morphological abnormalities as manifested by a reduction of the overall number of cells as well as a change in normal cellular morphology (i.e., some cells appeared to undergo apoptotic cell death with plasma membrane blebbing, cellular fragmentation, and shrinkage) (Figure 2C).

### 3.3. Reactive Species Generation and Cellular Internalization following Exposure to Subtoxic Concentrations of the Nanoparticles

**Figure 3:**
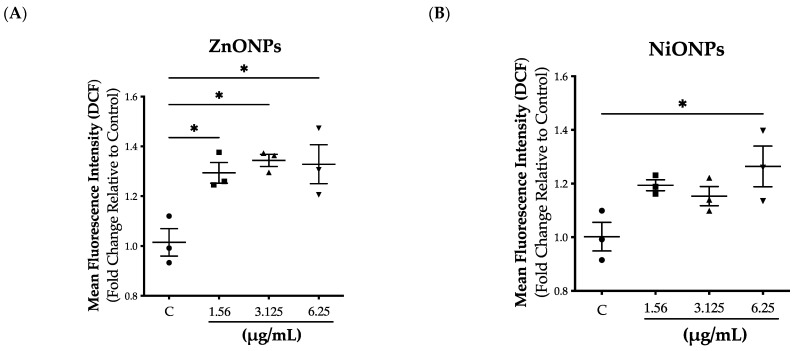
**Intracellular reactive oxygen species generation following exposure to subtoxic concentrations of nanoparticles.** Reactive oxygen species (ROS) generation following exposure to (**A**) ZnONPs or (**B**) NiONPs were measured using a ROS-sensitive fluorescent probe (dichlorofluorescin diacetate, H2DCFDA). Experiments were independently repeated at least three times (*n* ≥ 3), and statistical significance (*) indicates the difference between the treatment groups with *p*-values equal to or smaller than 0.05 (*p* ≤ 0.05). Circle: Control; square: 1.56 µg/mL; triangle up: 3.125 µg/mL; triangle down: 6.25 µg/mL.

Due to the large surface-to-volume ratio of nanoparticles, they are typically associated with reactive species generation, and this represents a major pathway driving their toxicity [31]. Therefore, we measured the levels of ROS following exposure to both nanoparticles at subtoxic concentrations (1.56–6.25 µg/mL). Our data demonstrated that cell exposure to both nanoparticles was associated with ROS generation (Figure 3A,B). However, the data suggests that exposure to ZnONPs induced higher ROS levels compared to NiONPs (Figure 3A,B).

**Figure 4:**
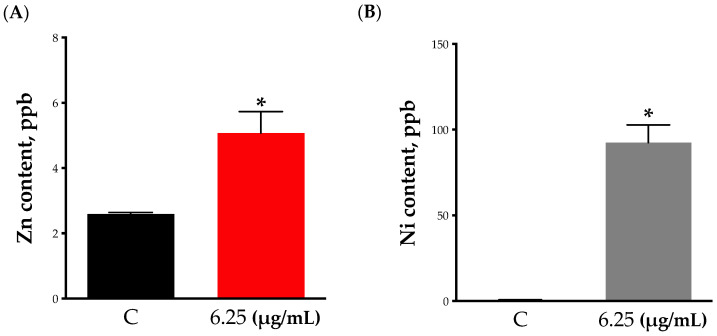
**Cellular internalization of nanoparticles.** The cell-internalized nanoparticles were evaluated by inductively coupled plasma mass spectrometry (ICP-MS). Cells were exposed to (**A**) ZnONPs (red bars) or (**B**) NiONPs (grey bars) for 24 h in serum-free cell culture media at 6.25 µg/mL. Experiments were independently repeated at least three times (*n* ≥ 3), and statistical significance (*) indicates the difference between the treatment group (black bars) with *p*-values equal to or smaller than 0.05 (*p* ≤ 0.05).

To gain some insights into the interaction between nanoparticles and cells, and whether toxicity is driven via cellular internalization of nanoparticles, we measured the concentrations of internalized nanoparticles using inductively coupled plasma mass spectrometry (ICP-MS). Our results showed that 24 h exposure to nanoparticles resulted in the cellular internalization of both nanoparticles (Figure 4A,B). However, exposure to NiONPs resulted in significantly higher cellular internalization in comparison with ZnONPs (i.e., ~100 vs. 5 ppb). These results suggest that ZnONP-mediated biointeraction is potentially driven at the cell membrane level.

### 3.4. Cellular Activation following Exposure to Subtoxic Concentrations of Nanoparticles

**Figure 5:**
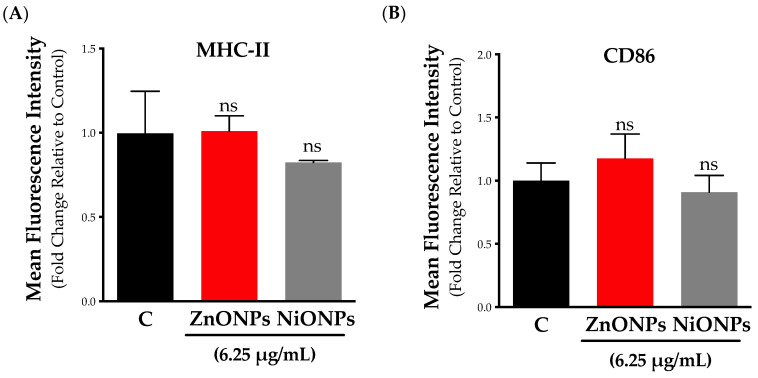
**Cellular activation following exposure to subtoxic concentrations of nanoparticles.** Cell activation was evaluated based on the surface expression markers (**A**) MHC-II and (**B**) CD86 as measured by flow cytometry. Cells were exposed to ZnONPs (red bars) or NiONPs (grey bars) for 24 h in serum-free cell culture media at 25 µg/mL. Experiments were independently repeated at least three times (*n* ≥ 3), and statistical significance (*) indicates the difference between the treatment groups (black bars) with *p*-values equal to or smaller than 0.05 (*p* ≤ 0.05) (Supplementary Figure S1).

It is well established that macrophages can be activated upon exposure to a plethora of stimuli, including pathogens and pathogenic components (e.g., LPS), as well as environmental toxicants (e.g., particulate matter, asbestos, etc.) [32]. As part of the characterization of ENM biological and toxicological responses, we sought to assess cellular activation following exposure to subtoxic concentrations of nanoparticles. One approach to assess macrophage activation is by measuring the increase in surface protein expression levels (e.g., MHC-II, CD86, CD68, etc.). Our results showed that neither ZnONPs nor NiONPs treatment at subtoxic concentration significantly changed the expression of the surface markers MHC-II and CD86 (Figure 5A,B).

### 3.5. Functional Assessment following Exposure to Subtoxic Concentrations of Nanoparticles

**Figure 6:**
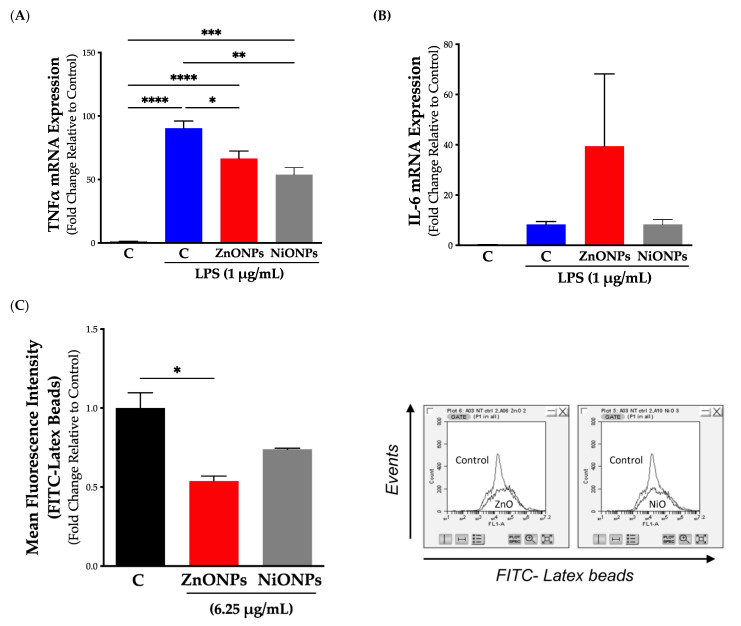
**Cellular functional capacity following exposure to subtoxic concentrations of nanoparticles.** Cell functional capacity was evaluated following exposure to nanoparticles at subtoxic levels. Cells were exposed to ZnONPs (red bars) or NiONPs (grey bars) at 6.25 µg/mL for 24 h in serum-free cell culture media and then were exposed to LPS (1 µg/mL) for 2 h after which (**A**) TNFα or (**B**) IL-6 inflammatory gene expression was measured by qPCR. (**C**) Cell phagocytic capacity was evaluated based on cellular uptake of latex beads coated with fluorophore-labeled rabbit IgG (the left panel shows the quantification of mean fluorescence intensity (MFI) of the FITC-labeled beads and the right panel shows a representative histogram). Experiments were independently repeated at least three times (*n* ≥ 3), and statistical significance indicates the difference between treatment groups (black bars) with *p*-values equal to or smaller than 0.05 (* *p* ≤ 0.05; ** *p* ≤ 0.01; *** *p* ≤ 0.001; **** *p* ≤ 0.0001).

One important parameter to evaluate following exposure to ENMs is cell functional capacity. It is established that macrophages respond to pathogens and foreign substances via the activation of different cellular processes, including the activation of the inflammatory response [26]. Evaluation of inflammatory response upon challenging with known stimuli is key to assessing cell functional competence. Our data showed that cell pre-exposure to nanoparticles resulted in changes in LPS-induced inflammatory gene expression. Specifically, pre-exposure to ZnONPs for 24 h reduced LPS-induced activation of TNFα gene expression and demonstrated a trend increase in IL-6 gene expression compared to non-exposed control (Figure 6A). Pre-exposure to NiONPs was also associated with a reduction in LPS-induced TNFα gene expression; however, there was no significant change in LPS-induced IL-6 gene expression compared to non-exposed control (Figure 6B).

One critical functional assessment of macrophages following exposure to ENMs is their capacity for phagocytosis. Indeed, a dysregulated phagocytic function could be associated with compromised immune function or excessive immune responses (e.g., autoimmune) [24]. Our results demonstrated that exposure to subtoxic concentrations of ZnONPs for 24 h was associated with reduced capacity for phagocytosis as measured by the uptake of latex beads coated with fluorophore-labeled rabbit IgG, while exposure to subtoxic concentrations of NiONPs showed a trend reduction in the cell phagocytic capacity (Figure 6C).

### 3.6. Cell Metabolomic Profile following Exposure to Subtoxic Concentrations of ZnONPs and NiONPs

**Figure 7:**
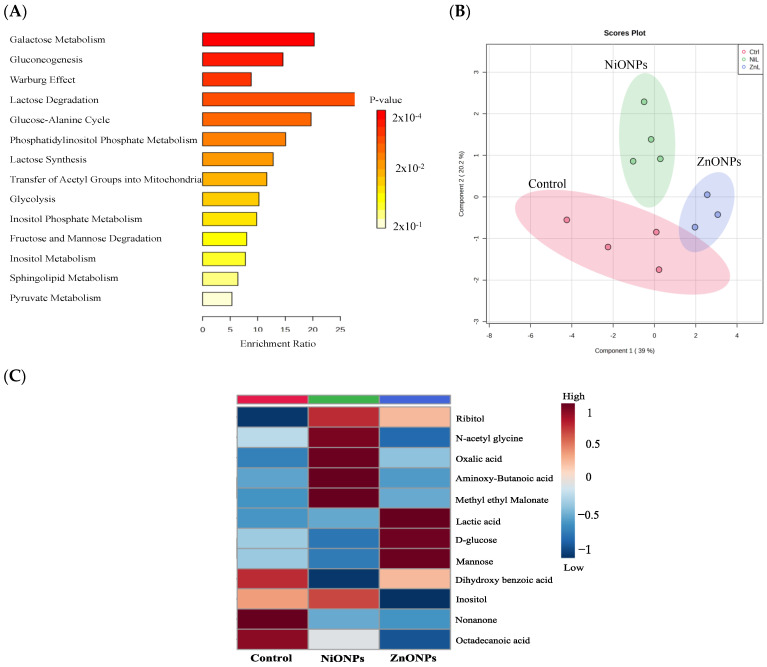
**Cellular metabolic changes following exposure to subtoxic concentrations of nanoparticles.** (**A**) Enrichment analysis showing the association between identified metabolites and metabolic pathways. (**B**) PLS-DA demonstrating the segregation and clustering of metabolic changes induced by nanoparticle treatment. (**C**) Heatmap illustrating the average group changes for each metabolite among treated groups. Experiments were independently repeated at least three times (*n* ≥ 3).

Previous studies have shown that exposure to inorganic ENMs such as silver, gold, titanium dioxide, and copper nanoparticles was associated with disruption of the primary metabolic pathways [33,34,35,36]. However, whether exposure to subtoxic concentrations of nanoparticles may alter cell metabolomic profile is yet to be determined. Therefore, we performed metabolomic profiling to assess changes in metabolite levels following exposure to subtoxic concentrations of ZnONPs or NiONPs. The results showed that the identified metabolites were linked to essential metabolic pathways involved in cell energetics and metabolism, including gluconeogenesis, glycolysis, and galactose metabolism (Figure 7A). We used the partial least-squares discriminant analysis (PLS-DA) to evaluate the impact of nanoparticle treatment on metabolite levels. The PLS-DA figure demonstrates clear clustering and separation of metabolites following exposure to subtoxic concentrations of ZnONPs or NiONPs (Figure 7B). Such results indicate that treatment with either ZnONPs or NiONPs drives a unique metabolic change. Treatment with ZnONPs resulted in an increase in the relative concentration of several metabolites such as lactic acid, mannose, and D-glucose, indicating an alteration in relevant pathways such as gluconeogenesis, lactose synthesis and degradation, and mannose degradation, while it decreased the relative concentration of other metabolites including inositol, 2-nonanone, and octadecanoic acid, which are essential components of inositol metabolism (Figure 7C). On the other hand, treatment with NiONPs was associated with a different pattern of changes in the concentration of metabolites compared to ZnONPs. For instance, it resulted in an increase in the relative concentration of N-acetyl glycine, oxalic acid, aminobutanoic acid, methyl ethyl malonate, and inositol, which are key parts of the inositol metabolism as well as in regulating energy production in the mitochondria (Figure 7C). There were no apparent changes in the levels of lactic acid, mannose, and D-glucose following treatment with NiONPs (Figure 7C).

## 4. Discussion

Ensuring the safe use of nanotechnology and ENMs remains the ultimate outcome of nanotoxicological research. The development of inorganic ENMs has been growing over the past years due to their unique inherent properties (e.g., electromagnetic, optical, mechanical, etc.) and high tuneability of their physicochemical properties (e.g., size, shape, surface charge, etc.) [31]. With the wide utilization and incorporation of inorganic ENMs in consumer and biomedical products, increased exposure may lead to adverse health outcomes [2]. Exposure to inorganic ENMs is often reported to be associated with toxicity at high exposure concentrations [31]. Nevertheless, whether exposure to inorganic ENMs at low and subtoxic concentrations (which is typically encountered in real life, e.g., consumer products) would be associated with adverse cellular consequences remains to be fully understood [1,4]. In this study, we evaluated potential adverse cellular responses following exposure to subtoxic concentrations of ZnONPs and NiONPs at the functional and metabolomic levels in a macrophage-like cell model.

Characterization of basic ENM toxicity, including dose–response cell viability, ROS generation, and nanoparticle internalization into cells, is important for any nanotoxicological study. Exposure to inorganic ENMs has often been reported to be associated with concentration-dependent toxicity across different tissues and cell types [8]. Similarly, our results demonstrated concentration-dependent toxicity following exposure to both nanoparticles. However, exposure to ZnONPs resulted in a sharp drop in cell viability at a concentration of 12.5 µg/mL and in almost complete loss of viability at higher concentrations, while exposure to NiONPs demonstrated a rather gradual reduction of cell viability with increased concentrations. Based on the viability study, a NOAEL of 6.25 µg/mL was used in subsequent experiments to assess any associated adverse response following a 24 h exposure to the nanoparticles. It is worth mentioning here that exposure to both nanoparticles at subtoxic concentrations (i.e., at or below 6.25 µg/mL) increased mitochondrial activity. This has been reported before in response to environmental exposures, including ENMs (wide range of compositions and physicochemical properties) [29,37,38,39,40]. Such an outcome may indicate that cells are responding to the insult by activating cellular pathways involved in the detoxification processes. It is also important to mention that nanoparticles may interfere with colorimetric assays [41]. However, we believe that this was not the case since there was a biphasic response following exposure to the nanoparticles (i.e., no consistent outcome with increasing concentrations). Also, our phase-contrast data was used to confirm the MTT assay results.

Reactive oxygen species generation following exposure to inorganic ENMs represents an established paradigm driving ENM cytotoxicity [42,43]. Our findings showed that exposure to both nanoparticles at subtoxic concentrations was associated with ROS generation. However, exposure to ZnONPs appeared to have induced higher levels of ROS. It is worth mentioning that ENM-induced ROS generation can be mediated at the cell membrane level or following ENM internalization into cells [15]. In addition, the assessment of nanoparticle internalization is key to gaining insights into ENM-mediated biointeractions [43]. Previous literature has shown that toxicity of inorganic ENM is mainly attributed to cellular internalization and consequent disruption of organelle function (e.g., lysosomes, mitochondria, etc.) [10]. Interestingly, our results showed that exposure to ZnONPs was associated with minimal cellular internalization, whereas exposure to NiONPs resulted in significant cellular internalization. We speculate that based on these results, ZnONP-induced biological outcomes are mediated via a cell membrane mechanism, whereas those induced by NiONPs are rather mediated through cellular internalization and accumulation into intracellular organelles [10]. Another possible explanation is that ZnONPs are being removed from the cells via cell efflux pathways rapidly after internalization. Such observed differences in cellular internalization could be attributed to differences in nanoparticle physicochemical properties, including size and surface charge (i.e., the smaller size and larger surface negative charge of NiONPs might have led to increased cellular interaction and internalization) [11].

The immune system, and specifically innate immune cells, are critical for the elimination of foreign bodies, including ENMs. Previous studies have established the fundamentals of immunotoxicity associated with ENM exposure, including the role of material composition and physicochemical properties (e.g., size, surface charge, shape, aspect ratio, surface functionalization, etc.) [14,17]. However, most of the previous research was carried out at high and unrealistic exposure levels [14,17]. Importantly, accumulating literature has demonstrated that exposure to inorganic ENMs at subtoxic concentrations may yet be associated with adverse cellular consequences and exacerbation of cellular injury [21,22,40,44,45,46,47]. For instance, it has been shown that macrophage exposure to a wide range of inorganic ENMs could result in functional alteration, such as changes in inflammatory gene expression and cell functional capacity upon challenging with pathogens or pathogenic components (e.g., LPS). Such a response was evident even in the absence of direct cellular toxicity or activation of cell stress and inflammatory responses [21,46]. For instance, a previous report has found that exposure to amorphous silica or superparamagnetic iron oxide nanoparticles (SPIONs) at 25 µg/mL for 24 h resulted in dysregulation of the cell activation response upon challenging with LPS or *Streptococcus pneumoniae* [21]. Another study has confirmed such findings in vivo, demonstrating that exposure to inorganic ENMs could increase susceptibility to lung infections as a result of modulating the macrophage function [46]. Despite the absence of apparent toxicity, these findings have been reported at relatively high exposure levels (e.g., 25 µg/mL). Therefore, an important question remains to be answered, that is, whether exposure to relatively low and subtoxic concentrations of inorganic ENMs (which typically produce toxicity at high exposure concentrations) could result in adverse cellular outcomes. The results of our study suggest that exposure to both nanoparticles at a relatively low and subtoxic concentration (i.e., 6.25 µg/mL) results in a modulation of LPS-mediated activation of inflammatory gene expression, including TNFα and IL-6. Such results are in agreement with previous studies confirming that exposure to ENMs at subtoxic concentrations may yet be associated with a detrimental impact on the cell functional capacity [21,46].

Earlier studies have demonstrated that exposure to inorganic ENMs, including high concentrations of ZnONPs, was associated with immunotoxicity, including a reduction in cell phagocytic capacity [22,40,44,46]. This is not surprising as overt cellular toxicity could be manifested in reduced cellular function. Most importantly, it has been previously shown that in vitro and in vivo exposure to subtoxic concentrations of SPIONs resulted in a significant reduction in macrophage phagocytic function [22,40,44,46]. We showed in a previous study that macrophage exposure to silver nanoparticles at relatively high but non-toxic concentrations (i.e., 50 µg/mL) was associated with a disruption in cell functional capacity, including changes in cell phagocytic ability [22]. In a similar fashion, the results in this report demonstrated that exposure to subtoxic concentrations of ZnONPs was associated with a reduced phagocytic function, and exposure to subtoxic concentrations of NiONPs appeared to have a trend reduction of the cell phagocytic function. Such findings are consistent with previous reports confirming and emphasizing that lack of direct and overt toxicity (e.g., cell viability, inflammatory response, oxidative stress, etc.) at subtoxic exposure to ENMs does not necessarily mean a lack of adverse cellular outcomes [21,22,40,44,46,47]. Additionally, the findings in this report also indicate that exposure to subtoxic levels of those ENMs with recognized toxicity at high exposure levels may still be associated with adverse health outcomes, particularly at the cell functional level. Future studies are warranted to investigate the underlying molecular mechanisms driving such modulation of cellular function following exposure to subtoxic levels of ENMs, including the impact of ion release (particularly for ENMs such as ZnONPs), as well as to unravel novel and sensitive biomarkers of exposure and toxicity. Interestingly, one previous study has shown a correlation between the propensity for inorganic ENMs to generate ROS and activate heme oxygenase 1 (hmox-1, a redox-sensitive protein) and reprogramming macrophage activation [46].

Omics tools such as transcriptomics, metabolomics, and proteomics enable the assessment of global alterations in gene expression, metabolite level, and protein expression, respectively. Such tools are key to understanding ENM-mediated biointeraction, cell biochemical state, and identification of novel biomarkers of exposure and toxicity following exposure to ENMs [27,28,48]. It is worth mentioning that previous studies have shown that exposure to a wide range of inorganic ENMs, including titanium dioxide, gold, silver, and copper nanoparticles, could be associated with perturbations of basic metabolic pathways such as those involved in bioenergetics, biosynthesis, and redox reactions in different experimental models [33,34,35,36]. Furthermore, it has been demonstrated that exposure to ENMs could disturb key cellular pathways, including apoptosis, ferroptosis, redox homeostasis, energy metabolism, mitochondrial function, and inflammatory responses [49,50,51]. To the best of our knowledge, this is the first study that assessed metabolomic changes at low and subtoxic concentrations of ZnONPs and NiONPs. The focus of this study was not to identify specific mechanisms of toxicity or do a comprehensive metabolomics study but rather to gain some insight into whether exposure to ENMs at subtoxic concentrations may influence metabolite clustering. Our findings demonstrated that exposure to each type of nanoparticle, even at subtoxic concentrations, was associated with a distinct clustering and separation of the measured metabolites compared to control. For example, there was an increase in several metabolites following exposure to ZnONPs but not NiONPs, including lactic acid, mannose, and D-glucose. The data also suggest that the changes are mainly involved in cellular bioenergetic metabolism. It would be difficult to explain the relevance of such metabolite changes at this stage as this is a small dataset. However, despite the technical limitations in this study, the findings suggest that it would be key to carry out a larger metabolomics study to characterize ENM-induced biointeractions at subtoxic exposure levels. Interestingly, a recent report has demonstrated ‘metabolomic signatures’ that could be used to assess the safety of inorganic ENMs as well as the degree of toxicity [52]. Whether such changes in metabolite levels reported in this study could serve as biomarkers or signatures of exposure and/or toxicity need to be validated in the future. Also, the current study did not compare the responses of nanoparticulate vs. released ions; however, we speculate that such responses are mainly driven by the nanoparticulate form of the metals as one recent study has indicated a particulate-specific effect vs. their released ions in ENM-induced metabolic changes [53]. The distinct metabolic disorders caused by various ENMs imply that ENMs have a unique molecular mechanism(s) of toxicity and hence, emphasize the importance of using metabolomics as a key tool for investigating the fundamental mechanisms of toxicity associated with exposure to ENMs [33,34,35,36]. Together, these data suggest a unique metabolic fingerprint following exposure to each type of nanoparticle. Therefore, carrying out an extensive metabolomics study following exposure to low and subtoxic ENM levels may identify sensitive metabolic alterations and explain potential adverse responses, even at concentrations that appeared to be safe by conventional toxicological endpoints.

## 5. Conclusions

In sum, the findings of this study suggest that exposure to inorganic ENMs may yet be associated with adverse health outcomes, particularly on the innate immune system (e.g., compromising the cell functional capacity), even at concentrations that are not associated with toxicity as per conventional toxicological endpoints. This study also demonstrates that the cell metabolome could be used as a key tool in the assessment of potential ENM-associated adverse cellular responses at low and subtoxic concentrations. The data reported in the study emphasizes the importance of assessing the safety of ENMs at low and subtoxic exposure levels, particularly at the functional levels. Furthermore, the utilization of sensitive functional assays and metabolomic tools may become key in the future assessment of the ENM safety profile.

## Figures and Tables

**Table 1:** Characterization of ZnONPs and NiONPs in dd water and cell culture media (DMEM).

Nanoparticle—Vehicle	Hydrodynamic Size (nm)	Surface Charge (ζ) (mV)
ZnONPs—ddH_2_O	336.8 ± 25.5	−17.9 ± 2.1
ZnONPs—DMEM	427.7 ± 18.9	−1.5 ± 0.2
NiONPs—ddH_2_O	217.6 ± 11.3	−36.3 ± 1.4
NiONPs—DMEM	384.7 ± 21.3	−14.9 ± 1.9

## Data Availability

Not applicable.

## Supplementary Materials

The following supporting information can be downloaded at: https://www.mdpi.com/article/10.3390/toxics11080674/s1, Figure S1: Flow cytometry histograms of Figure 5.
