# Supplementary figures and images for: Adverse Responses following Exposure to Subtoxic Concentrations of Zinc Oxide and Nickle Oxide Nanoparticles in the Raw 264.7 Cells
Source: Toxics. 2023 Aug 6;11(8):674. doi: 10.3390/toxics11080674 (PMC10459918; doi:10.3390/toxics11080674)

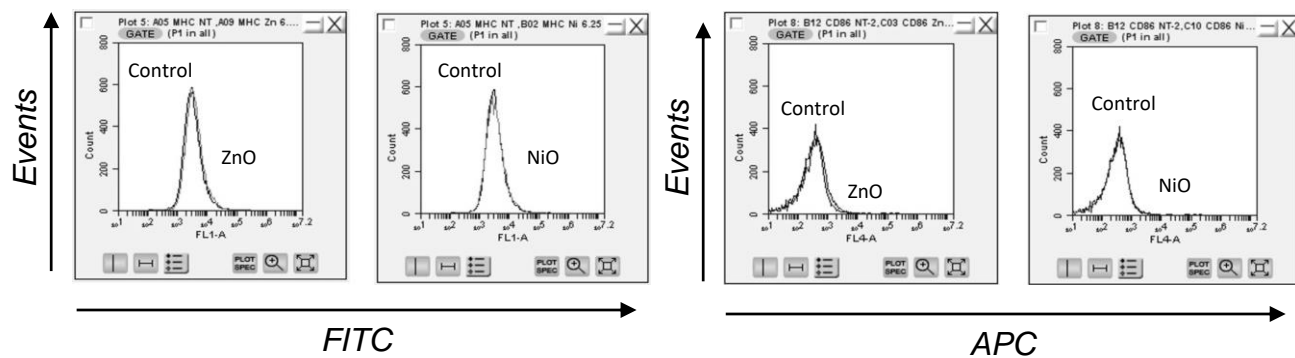

Figure S1

Supplement: Supplementary file 1 [file toxics-11-00674-s001.zip › toxics-2439522-supplementary.pdf]
